# The efficacy of L-carnitine in patients with nonalcoholic steatohepatitis and concomitant obesity

**DOI:** 10.1186/s12944-023-01867-3

**Published:** 2023-07-12

**Authors:** Natalia Zakharova, Chenguang Luo, Raisa Aringazina, Vadim Samusenkov

**Affiliations:** 1 Department of Chemistry, I.M. Sechenov First Moscow State Medical University (Sechenov University, Trubetskaya str., 8-2, Moscow, 119991 Russian Federation; 2 Department of Hospital Therapy named after Academician G.I. Storozhakov of the Medical Faculty, Pirogov Russian National Research Medical University, Ostrovityanova str., 1 , Moscow, 117997 Russian Federation; 3 Department of Internal Diseases № 1, Non-Commercial Joint-Stock Society “West Kazakhstan Marat Ospanov Medical University”, Aleksey Maresyev str, Aktobe, 030019 Kazakhstan; 4 Department of Prosthetic Dentistry, I.M. Sechenov First Moscow State Medical University (Sechenov University), Trubetskaya str., 8-2, Moscow, 119991 Russian Federation

**Keywords:** Nonalcoholic steatohepatitis and concomitant obesity, The effects of L-carnitine on liver tests, L-carnitine for the management of hyperlipidemia, Improving insulin resistance in steatohepatitis, Carbohydrate metabolism

## Abstract

**Background:**

In light of the high prevalence of nonalcoholic fatty liver disease and obesity, treatment options for nonalcoholic steatohepatitis are of particular interest. The purpose of the study is to assess the efficacy of L-carnitine and its effects on the functional state of the liver, as well as on lipid and carbohydrate metabolism in patients with nonalcoholic steatohepatitis and concomitant obesity.

**Methods:**

People in the control group followed a hypocaloric diet and received 1 tablet of simvastatin 20 mg once a day and 2 capsules of essential phospholipids 600 mg three times a day for 90 days. People in the experimental group followed a hypocaloric diet and received 1 tablet of simvastatin 20 mg once a day and L-carnitine 10 mL orally two times a day for 90 days.

**Results:**

L-carnitine normalized the blood lipid profile of subjects, as demonstrated by a significant decrease in the blood levels of total cholesterol, triglycerides, low-density lipoproteins, atherogenic index, and insulin resistance. The use of L-carnitine in patients with nonalcoholic steatohepatitis and concomitant obesity contributes to the steady reduction of the main clinical and biochemical symptoms of nonalcoholic steatohepatitis.

**Conclusions:**

L-carnitine produces positive effects on the blood lipid profile and carbohydrate metabolism.

## Introduction

### Scientific relevance

Nonalcoholic fatty liver disease (NAFLD) is one of the most common liver diseases. The burden of NAFLD is increasing worldwide due to the obesity epidemic and sedentary lifestyle [1–3]. The prevalence of NAFLD in the general population ranges from 10 to 24% [2–5]. The highest rates are in South America – 17–33% [2, 4, 6, 7], the Middle East – 6–32% [3, 8–10], Asia – 15–30% [2, 3, 11–14], the USA – 27–34% [1–3, 15–19], and Europe – 23–31% [2, 3, 20]. Such a wide range for different regions is due to several reasons. These are different criteria for selecting patients, different diagnostic methods and nutritional characteristics, as well as dissimilar lifestyles.

The diagnostic process of NAFLD can be intricate, typically relying on a combination of clinical, laboratory, instrumental, and histological data. According to the recommendations provided by the European Association for the Study of the Liver (EASL), the following criteria are employed for the diagnosis of NAFLD:

Exclusion of other causes of hepatic steatosis: The diagnosis of nonalcoholic steatohepatitis (NASH) necessitates the exclusion of alternative causes of liver fat accumulation, such as alcoholic liver disease, secondary steatosis (e.g., due to medication or genetic disorders), and hereditary hepatic steatosis.

Presence of hepatic steatosis: The diagnosis of NASH requires the confirmation of hepatic steatosis. This can be determined through noninvasive methods, including ultrasonography, computed tomography, or magnetic resonance imaging of the liver.

Exclusion of alcoholic liver disease: It is essential to exclude significant alcohol consumption or alcoholic liver disease as the cause of hepatic steatosis. This may require a detailed medical history, patient interviews, and/or specific laboratory tests.

Presence of inflammation and/or fibrosis: The diagnosis of nonalcoholic steatohepatitis (NASH) requires confirmation of the presence of inflammation and/or fibrosis in the liver. This can be assessed through liver biopsies or noninvasive methods, such as FibroScan, measurement of alanine aminotransferase (ALT) and aspartate aminotransferase (AST) levels in the blood, and the calculation of fibrosis indices (e.g., NAFLD Fibrosis Score or Fibrosis-4 Index).

Absence of alternative causes of inflammation and fibrosis: The diagnosis of nonalcoholic steatohepatitis (NASH) requires the exclusion of alternative causes of liver inflammation and fibrosis, such as viral hepatitis, autoimmune liver diseases, and genetic disorders.

The prevalence of NAFLD is high in people with insulin resistance (IR), who may suffer from obesity, type 2 diabetes mellitus, dyslipidemia, and metabolic syndrome [1–3, 16, 18].

According to studies involving NAFLD patients, approximately 30% of hepatic steatosis cases evolve into steatohepatitis [3], which inevitably progresses to fibrosis and later to cirrhosis [21]. The latter is regarded as the ‘point of no return’ and is associated with high mortality [15, 21]. In addition, HCC (hepatocellular carcinoma) may develop as a complication of cirrhosis in patients with NAFLD. Interestingly, there are reports of HCC as a rare complication in people with NAFLD and without cirrhosis [22]. At the same time, NASH diagnosis is a risk for extrahepatic cancers, mainly in older patients, for example, bladder cancer, as evident in [23]. A recent global meta-analysis showed that the annual incidence rate of NASH was 5.3 per 1,000 person-years [24]. Mortality rates of HCC were 0.25% and 2.3% after a follow-up of 8.3 and 13.7 years, respectively [25]. Etiopathogenetic therapy for NAFLD/NASH has not been elaborated on thus far. There is no specific pharmacological treatment for NAFLD approved by the U.S. Federal Food and Drug Administration (FDA) or the European Medicines Agency (EMA) [26].

First, NASH therapy should aim to eliminate the factors contributing to disease development and progression, reduce hepatocyte damage, prevent steatohepatitis progression to fibrosis and cirrhosis, improve tissue sensitivity to insulin, and manage NASH-related metabolic disorders [4, 5, 26, 27]. It is well known that TM6SF2 and other genes are involved in the development of NASH [28–30].

Currently, researchers are searching for new drugs for NASH therapy. A large variety of drugs are in development, and studies have addressed some of them [31]. Ursodeoxycholic acid (ursosan) is widely used for the treatment of patients with NAFLD. The theoretical justification for its use in nonalcoholic steatohepatitis is its anti-apoptotic, immunomodulatory, and hypocholesterolemic effects confirmed by clinical data. In a randomized double-blind controlled trial by Ratziu et al. [32], UDCA (Ursodeoxycholic Acid) therapy at a dose of 28–35 mg/kg per day significantly reduced ALT activity compared to placebo: 28.3 and 1.6%, respectively (*P* < 0.001). Some experts advise using natural products in the diet to prevent NASH due to its connection with obesity, although the effectiveness of this prevention remains debatable [33].

Obesity has been linked to metabolic syndrome, type 2 diabetes, and nonalcoholic fatty liver disease (NAFLD). Obesity causes a decrease in growth hormone (GH) levels and an increase in insulin levels. Long-term GH treatment increased lipolytic activity as opposed to decreasing insulin sensitivity. Nonetheless, it is possible that short-term GH administration had no impact on insulin sensitivity [34].

Aerobic exercise and vitamin E supplementation can improve HFD-induced NAFLD in rats by regulating the AMPK pathway and reducing oxidative stress [35].

An agent that can alleviate the main clinical and biochemical symptoms of NASH and prevent its progression is L-carnitine. This compound has antioxidant, lipid-lowering, cytoprotective, and anabolic effects. It participates in energy metabolism, phospholipid synthesis, and fat catabolism. These processes occur by the stimulation of fatty acid oxidation with their subsequent transportation to mitochondria, resulting in a decrease in the lipid contents of tissues [5, 36–41]. L-carnitine helps to reduce the level of lipids in the blood serum with a high-fat diet and reduces their content in the liver. This is due to the increased oxidation of fatty acids, although the role of L-carnitine as a powerful antioxidant has not been confirmed [42]. An earlier study showed the role of L-carnitine in reducing insulin secretion and improving glucose metabolism [43]. Although the mechanisms of L-carnitine’s effect on lipid and carbohydrate metabolism have been sufficiently studied, there is a need for a broader evidence base to introduce this compound into the diet. This study aims to supplement the existing information and provide more detailed research on the effects of taking L-carnitine compared with taking phospholipids on the activity of liver enzymes and carbohydrate metabolism [44, 45]. The purpose of the current study is to assess the efficacy of L-carnitine and its effects on the functional state of the liver, as well as on lipid and carbohydrate metabolism in patients with NASH and concomitant obesity.

## Materials and methods

### Sampling

The study involved 90 patients with NASH and concomitant obesity examined for data comparison purposes. The mean age of the patients was 40.6 ± 6.38 years. Among the examined patients with NASH and concomitant obesity, there were 56 men (62.2%) and 34 women (37.8%). Two study groups were formed to assess the treatment efficacy. The inclusion of patients in both groups was random. The randomization procedure was implemented in the following manner. Simple random sampling was employed, ensuring that each patient had an equiprobable opportunity to be assigned to either of the two groups. For instance, computer-based programs using random number generators were used to allocate patients randomly to their respective groups. The randomization process was administered by an independent party, distinct from the researchers, to mitigate the potential influence of biases or preferences during a patient assignment.

Table 1 presents more detailed data.

**Table 1:** Demographic data of patients from groups 1 and 2

Group	The number of men, age	The number of women, age	The degree of obesity, minimum-maximum, average	Waist-to-height ratio (WHtR)	The severity of the cytolytic syndrome
1	29	16	31–45, 36.0	0.827 (man) 0.874 (woman)	Necrobiosis (reversible)
2	27	18	31–44, 35.0	0.828 (man) 0.875 (woman)	Necrobiosis (reversible)

### Study design

Additionally, the patients were randomized by age, sex, obesity grade, and the severity of cytolytic syndrome. Subjects in the control group (group 1, 45 individuals) followed a hypocaloric diet and received 1 tablet of simvastatin 20 mg once a day and 2 capsules of essential phospholipids 600 mg three times a day for 90 days. Subjects in the experimental group (group 2, 45 individuals) followed a hypocaloric diet and received 1 tablet of simvastatin 20 mg once a day and L-carnitine 10 mL orally two times a day for 90 days. Patients in this group did not receive phospholipids. In a study involving two equal groups (experimental and control), a placebo may not be necessary when assessing the effectiveness of a new therapeutic intervention compared to an already established standard treatment or absence of treatment. In such cases, the control group may consist of patients receiving standard treatment or receiving no treatment at all (no treatment group).

The use of a placebo in such a study may be ethically unacceptable. If the efficacy of the standard treatment has already been established, the use of a placebo can be unsafe or ethically inappropriate, as it may deprive patients of available and effective treatment.

Other studies also used 90 days as the period to study the effect of L-carnitine [46]. The diet included limiting fat intake to 25–30% of the total energy value; limiting the consumption of foods with a high cholesterol content (sausage, whole milk, dairy products, etc.) – no more than 300 mg per day, excluding fried dishes; eating foods with a high content of vitamins and natural prebiotics (fruit, topinambur, leek, artichoke).

### Therapeutic methods

In addition, all subjects followed a program designed to increase their physical activity levels: they took 40-minute walks at a moderate pace two times a day. The monitoring of patients’ walking time was remote and involved using a pedometer. The study is double-blind. The diet and recommendations from doctors predetermined the patients’ lifestyles.

The diagnosis of NASH was established according to the recommendations of the European Association for the Study of the Liver (EASL). The diagnosis of obesity was established based on the classification proposed by the World Health Organization (WHO) International Working Group on Obesity (1997) and in compliance with the guidelines of the European Association for the Study of Obesity (EASO). To clarify, obesity was diagnosed in individuals with a body mass index (BMI) of 30 kg/m^2^ or greater. The basis for determining the stage of obesity was the generally accepted calculation according to the following formula:

BMI = body mass (in kilograms)/(height (in meters))^2,

where body mass is measured in kilograms and height is measured in meters. This calculation did not show significant differences between the groups according to the degree of BMI.

Possible development mechanisms of steatosis are a decreased synthesis of very-low-density lipoproteins (VLDL) and an increased synthesis of hepatic triglycerides (possibly due to a decrease in the oxidation of fatty acids or an increase in the amount of free fatty acids entering the liver). Inflammation can occur due to lipid peroxidation, which causes damage to cell membranes. These changes can stimulate the stellate cells of the liver, thereby leading to fibrosis. NASH can cause the development of cirrhosis and portal hypertension. Most patients have no symptoms. Nevertheless, some patients experience weakness, malaise, or discomfort in the upper right quadrant of the abdomen. Hepatomegaly develops in approximately 75% of patients. Severe liver fibrosis may cause splenomegaly. Typically, this is the first sign of portal hypertension. Patients with cirrhosis due to NASH may be asymptomatic and have no signs of chronic liver disease. The prognosis depends on the degree of fibrosis and is the only indicator that correlates with the mortality associated with liver damage and the need for liver transplantation [47]. The prognosis is challenging to predict, although patients with NAFLD who have NASH according to histology results and data indicating fibrosis are more likely to develop cirrhosis [48]. According to estimations, in 10% of patients with NAFLD, the disease progresses to liver cirrhosis within 20 years [47, 48]. Alcohol, as well as some drugs (for example, cytotoxic) and metabolic disorders, are associated with the progression of nonalcoholic steatohepatitis (NASH). A liver biopsy was previously performed to diagnose NASH. The stages of fibrosis varied from early (F0-F1) to significant (F2), pronounced (F3), and up the liver cirrhosis on the background of NASH (F4) [47, 48]. Among the patients in group 1, 38 had stage F0-F1 disease, while the rest had stage F2 disease. In group 2, there were 37 patients with the F0–F1 stage, and 8 people had the F2 stage. The later stages of fibrosis were not included in the study due to the severe pathological course of this disease at this stage. The placebo effect was replaced with phospholipids.

The study involved only individuals aged 30 to 50 who had verified diagnoses of NASH and obesity. The diagnosis of NASH depended on the results of liver enzyme assays. All study subjects signed a voluntary informed consent to participate in the study.

### Inclusion and exclusion criteria

Exclusion criteria included chronic alcohol abuse and alcohol consumption in clinically significant amounts (more than 70 units per week for women and more than 140 units per week for men). Candidates with autoimmune hepatitis, viral hepatitis B, C, and D, genetic liver diseases, toxic liver damage, liver cirrhosis, and concomitant decompensated or acute chronic diseases were considered ineligible. In addition, pregnant, breastfeeding and noncompliant patients were not allowed to participate.

### Tests and biochemical methods for assessing the functional status of the liver

The liver’s functional state was assessed based on the activities of hepatic transaminases – alanine aminotransferase (ALT) and aspartate aminotransferase (AST), alkaline phosphatase (ALP), γ-glutamyltransferase (GGT), blood levels of bilirubin and its fractions. In addition, a thymol test was performed. The blood lipid spectrum was investigated by measuring the contents of total lipids, triglycerides (TG), total cholesterol (TC), low-density lipoproteins (LDL), and antiatherogenic high-density lipoproteins (HDL). Moreover, the atherogenic index (AI) was calculated using the following formula: AI = TC–HDL/HDL. All of the above tests were performed at baseline (T0), on day 30 (T1), and day 90 (T2) after therapy.

Carbohydrate metabolism was evaluated by the blood levels of fasting and 2-hour postload glucose and fasting insulin levels. The IR degree was determined based on the HOMA-IR index calculated using the HOMA Calculator Version 2.2 Diabetes Trials Unit University of Oxford (United Kingdom).

Since the study was conducted in a day hospital, patients with NASH had the opportunity to take medications. The same specialists carried out the sampling at the beginning of the study (T0) and on days 30 (T1) and 90 (T2). This approach aimed to exclude the error factor of different researchers. The changes in biochemical laboratory parameters reflecting the functional liver state, blood lipid spectrum, and carbohydrate metabolism were assessed over the treatment course. The study implied a significant effect of L-carnitine on the blood lipid profile in patients diagnosed with NASH in the experimental group. The expected effect of this was a decrease in clinical and biochemical symptoms in patients from the experimental group.

When interpreting the results of the thymol turbidity test (TT) as an indicator of liver dysfunction, caution should be exercised, and it should be noted that this test is outdated and less accurate than modern methods for assessing liver function. Currently, there are more reliable and precise methods for evaluating liver function, such as measuring liver enzymes (e.g., ALT and AST), biochemical tests (e.g., measuring bilirubin, protein, and other markers), and noninvasive methods, including liver elastography and FibroScan, which allow for the assessment of liver fibrosis without the need for biopsy.

### Statistical analyses

Statistical processing of the study results was performed using parametric (Student’s t test, Fisher’s F test) and nonparametric (Mann‒Whitney U test, Wilcoxon’s T test) statistical methods intended for the analysis of variance. Moreover, ANOVA (analysis of variance) was employed, using baseline values as a covariate, to compare within-group (within-group) variance with between-group (between-group) variance.

Normally distributed quantitative data are presented as the mean ± standard deviation (SD). A *P* value of 0.05 or less was considered statistically significant in all cases. Before starting the study, the sample size was calculated. Statistical and graphical analyses of the results were performed using Microsoft Excel 2013 (Microsoft, USA) and SPSS™ 17 packages.

### Ethics approval

The research was conducted in compliance with the ethical standards of the World Medical Association Declaration of Helsinki. The research was approved by the Local Ethics Committees of West Kazakhstan Marat Ospanov Medical University (Protocol No. 334). All study participants provided informed consent.

## Results

### The results of taking drugs in groups 1 and 2

According to the data collected on day 30 (T1 stage) from group 1, subjects who administered essential phospholipids, 24 (53.3%) patients had generalized weakness, 19 (42.2%) patients had heaviness in the right hypochondrium, 18 (40%) patients had flatulence, 13 (28.9%) patients experienced bitter taste in the mouth, 9 (20%) patients had nausea, 8 (17.8%) patients suffered from bowel disorders, and 3 (6.7%) patients had recurrent moderate pain in the upper right abdomen. As of day 90 (T2), there were no reports of nausea, pain in the upper right abdomen, or bowel disorders in group 1 subjects. However, generalized weakness persisted in 11 (24.4%) patients, moderate heaviness in the right hypochondrium in 9 (20%) patients, flatulence in 6 (13.3%) patients, and bitter taste in the mouth in 4 (8.9%) patients. In group 2, 7 (15.6%) patients reported flatulence, 11 (24.4%) noted heaviness in the right hypochondrium, and 14 (31.1%) mentioned generalized weakness. On T2, only 6 (13.3%) patients in group 2 complained of generalized weakness, 4 (8.9%) patients complained of heaviness in the right hypochondrium, and 3 (6.7%) patients complained of flatulence.

BMI significantly decreased in patients from group 2, who took L-carnitine. The decrease at 90 days was approximately 7% in group 2 and 3% in group 1. Thus, L-carnitine therapy had a more significant effect on reducing BMI in group 2 than in group 1 (*Р*≤0.05).

### The main biochemical indicators of liver function

Following an analysis of the main biochemical parameters of the functional liver state performed at T1 after the start of treatment, we found that a significant decrease in total bilirubin was observed only in group 2 subjects (1.65 times decrease, *P* < 0.05, F = 4.11). There was only a decreasing tendency in group 1 (*Р*>0.05, Table 2, F = 3.83). Significant changes in the contents of bilirubin were observed at the end of treatment. Thus, at T2 after the start of treatment, the levels of total bilirubin significantly decreased in group 1 and group 2 subjects by 2.1 (*P* < 0.05, F = 4.22) and 2.7 times (*P* < 0.05, F = 4.31), respectively, with a statistically significant intergroup difference (*P* < 0.05, F = 4.24).

**Table 2:** The results of taking drugs in groups 1 and 2 with nonalcoholic steatohepatitis and concomitant obesity, mean ± SD

Parameter	Group	T0 of treatment	T1 after the start of treatment	T2 after the start of treatment
Total bilirubin, µmol/L	1	41.72 ± 2.04*	30.42 ± 3.80*	19.82 ± 1.16 **
	2	41.5 ± 1.96*	25.1 ± 2.13**	15.47 ± 0.99**/***
ALT, U/L	1	80.31 ± 8.40*	50.62 ± 4.76*/**	31.67 ± 2.08 **
	2	79.60 ± 9.05*	41.29 ± 3.5*/**	21.33 ± 1.61**/***
AST, U/L	1	67.4 ± 5.22*	47.26 ± 3.18*/**	29.94 ± 1.73**
	2	67.75 ± 4.93*	38.47 ± 3.04*/**	22.5 ± 1.28**/***
GGT, U/L	1	60.54 ± 5.69*	52.6 ± 3.4*	38.2 ± 2.10**
	2	60.70 ± 5.93*	40.11 ± 2.02*/**/***	27.76 ± 1.34**/***
ALP, U/L	1	156.23 ± 9.17*	129.36 ± 7.44*	98.56 ± 5.62*/**
	2	157.06 ± 8.8*	101.22 ± 6.15*/**/***	76.09 ± 5.11**/***
Thymol test, conventional units	1	6.25 ± 0.18*	4.28 ± 0.20*/**	2.65 ± 0.13*/**
	2	6.30 ± 0.14*	3.59 ± 0.13*/**/***	1.93 ± 0.09**/***
**Reduction in cholesterol levels**, **%**	1 2	0 0	14–18 17–25	19–23 21–29

Note: ** – the difference is significant compared to the pretreatment value (*P* < 0.05); *** – the difference is significant compared to the posttreatment value obtained for group 1 subjects (*P* < 0.05)

Positive changes in the activities of the main cytolysis markers (ALT, AST) were observed in patients from both groups. Thus, by T1, the activity of ALT in group 1 subjects decreased by 1.6 times (*P* < 0.05, F = 4.10), and in group 2 subjects, it decreased by 1.93 times (*P* < 0.05, F = 4.16). As of T2, there was a 2.5-fold (*P* < 0.05, F = 4.35) decrease in group 1 subjects and a 3.7-fold decrease (*P* < 0.05, F = 4.91) in group 2 subjects, with a statistically significant intergroup difference (*P* < 0.05, F = 4.41). A similar trend was revealed for AST activity: at T1 after the start of treatment, there was a 1.4-fold decrease (*P* < 0.05, F = 4.15) in group 1 subjects and a 1.76-fold decrease (*P* < 0.05, F = 4.22) in group 2 subjects. By T2, the activity of AST decreased by 2.25 times (*P* < 0.05), F = 4.44 in group 1 subjects and by 3.0 times (*P* < 0.05, F = 4.67) in group 2 subjects, with a significant intergroup difference (*P* < 0.05, F = 4.52).

Furthermore, there was a decrease in the activities of GGT and ALP. Thus, by T1, group 1 subjects showed only a decreasing tendency (*P* > 0.05, F = 3.88), whereas group 2 subjects experienced a significant decrease in GGT (by 1.5 times, *P* < 0.05, F = 4.12) and ALP (by 1.55 times, *P* < 0.05, F = 4.17). By T2 after the start of treatment, the activities of GGT and ALP decreased by 1.6 (*P* < 0.05, F = 4.20) and 1.58 times (*P* < 0.05, F = 4.18) in group 1 subjects, while in group 2 subjects, they decreased by 2.2 (*P* < 0.05, F = 4.32) and 2.06 times (*P* < 0.05, F = 4.27), with a statistically significant (*P* < 0.05, F = 4.25) intergroup difference.

On T1 after the start of treatment, there was a 1.5-fold decrease (*P* < 0.05, F = 4.16) in thymol turbidity test values in group 1 subjects. At the same time, in group 2 subjects, the decrease was 1.75 times (*P* < 0.05, F = 4.22). As of T2, there were 2.4 (*P* < 0.05, F = 4.34) and 3.3-fold decreases (*P* < 0.05, F = 4.72), respectively, with a statistically significant intergroup difference (*P* < 0.05, F = 4.52).

### The blood lipid spectrum

Having explored the blood lipid spectrum, we noticed a positive trend in both comparison groups (Table 3, F = 4.18). Notably, by T1 after the start of treatment, TC decreased by 1.2 times (*P* < 0.05) in group 1 subjects and by 1.3 times (*P* < 0.05) in group 2 subjects. Next, TG decreased by 1.2 (*P* < 0.05) and 1.5 times (*P* < 0.05), LDL by 1.3 (*P* < 0.05) and 1.6 times (*P* < 0.05), and AI by 1.4 (*P* < 0.05) and 1.75 times (*P* < 0.05), respectively, with a statistically significant intergroup difference (*P* < 0.05). As of T2, TC decreased by 1.36 times (*P* < 0.05) in group 1 subjects and by 1.53 times (*P* < 0.05) in group 2 subjects. In addition, TG decreased by 1.77 (*P* < 0.05) and 2.2 times (*P* < 0.05), LDL by 1.65 (*P* < 0.05) and 1.9 times (*P* < 0.05), and AI by 2.1 (*P* < 0.05) and 2.8 times (*P* < 0.05), respectively, with a statistically significant intergroup difference (*P* < 0.05). In addition, there was an increase in the blood contents of antiatherogenic HDL. On T1, HDL levels increased by 1.16 (*P* < 0.05) and 1.28 times (*P* < 0.05) in groups 1 and 2, respectively, with a statistically significant intergroup difference (*P* < 0.05). On T2, there was a 1.36 (*P* < 0.05) and 1.47-fold increase (*P* < 0.05) in HDL levels, with a statistically significant intergroup difference (*P* < 0.05).

**Table 3:** The dynamics of changes in blood lipids observed during the course of treatment in patients with nonalcoholic steatohepatitis and concomitant obesity, mean ± SD

Parameter	Group	T0 of treatment	T1 after the start of treatment	T2 after the start of treatment
TC, mmol/L	1	7.04 ± 0.28	5.96 ± 0.14	5.17 ± 0.11**
	2	6.95 ± 0.24	5.50 ± 0.18**	4.51 ± 0.04**/***
TG, mmol/L	1	3.25 ± 0.06	2.73 ± 0.09**	1.82 ± 0.05**
	2	3.26 ± 0.05	2.21 ± 0.06 **/***	1.43 ± 0.03**/***
LDL, mmol/L	1	4.84 ± 0.03	3.75 ± 0.06**	2.94 ± 0.05*/**
	2	4.81 ± 0.03	3.07 ± 0.04**/***	2.49 ± 0.03**/***
HDL, mmol/L	1	0.98 ± 0.03 (Men: 0.96 ± 0.04; Woman: 0.94 ± 0.03)	1.14 ± 0.02** (Men: 1.16 ± 0.03; Woman: 1.12 ± 0.03)	1.31 ± 0.02*/** (Men: 1.33 ± 0.03; Woman: 1.29 ± 0.03)
	2	0.97 ± 0.02 (Men: 0.95 ± 0.03; Woman: 0.93 ± 0.04)	1.26 ± 0.01**/*** (Men: 0.97 ± 0.03; Woman: 0.93 ± 0.03)	1.42 ± 0.01**/*** (Men: 1.44 ± 0.04; Woman: 1.40 ± 0.03)
AI	1	6.3 ± 0.06*	4.54 ± 0.04*/**	2.95 ± 0.04*/**
	2	6.14 ± 0.08*	3.5 ± 0.05*/**/***	2.18 ± 0.03*/**/***

Note: ** – the difference is significant compared to the pretreatment value (*P* < 0.05); *** – the difference is significant compared to the posttreatment value obtained for group 1 subjects (*P* < 0.05)

The steatosis degree decreased to zero in 45 patients from group 2 and 39 patients from group 1. Consequently, patients from group 2 with L-carnitine administration experienced a more considerable effect in terms of improving liver function.

### Carbohydrate metabolism

In addition, group 2 subjects had pronounced positive changes in carbohydrate metabolism (Table 4, F = 4.28). Accordingly, by T1 after the start of treatment, fasting blood glucose significantly decreased in group 2 subjects (by 12.5%, *P* < 0.05). There was only a decreasing tendency at this time point in group 1 (*P* > 0.05). More dramatic changes were observed at T2: fasting blood glucose decreased by 9% (*P* < 0.05) in group 1 and by 14.6% (*P* < 0.05) in group 2, with a statistically significant intergroup difference (*P* < 0.05).

**Table 4:** The dynamics of changes in carbohydrate metabolism observed during the course of treatment in patients with nonalcoholic steatohepatitis and concomitant obesity, mean ± SD

Parameter	Group	T0 of treatment	T1 after the start of treatment	T2 after the start of treatment
Fasting glucose, mmol/L	1	5.77 ± 0.13*	5.63 ± 0.14*	5.25 ± 0.08**
	2	5.81 ± 0.15*	5.08 ± 0.09**/***	4.96 ± 0.05**/***
2-hour postprandial glucose, mmol/L	1	9.01 ± 0.32*	8.57 ± 0.24*	7.80 ± 0.14*/**
	2	9.05 ± 0.24*	7.96 ± 0.16*	7.31 ± 0.10**/***
Insulin, µU/mL	1	23.10 ± 2.41*	18.86 ± 2.33*	16.2 ± 1.32*
	2	23.15 ± 2.35*	15.17 ± 1.48*/**	10.02 ± 0.55**/***
HOMA-IR	1	3.02 ± 0.12*	2.47 ± 0.18 *	2.10 ± 0.15*/**
	2	3.03 ± 0.14*	1.82 ± 0.08 */**/***	1.29 ± 0.07**/***

Note: ** – the difference is significant compared to the pretreatment value (*P* < 0.05); *** – the difference is significant compared to the posttreatment value obtained for group 1 subjects (*P* < 0.05)

On T1, both comparison groups demonstrated only a tendency toward a decrease (*P* > 0.05) in blood glucose levels 2 h after eating. On T2,2-hour postprandial glucose significantly decreased by 12.8% (*P* < 0.05) in group 1 and by 19.1% (*P* < 0.05) in group 2, with a significant intergroup difference (*P* < 0.05).

By T1 after the start of treatment, the blood levels of insulin decreased by 1.53 times (*P* < 0.05) in group 2 subjects, while group 1 subjects had only a decreasing tendency (*P* > 0.05). As of T2, the blood levels of insulin decreased by 1.43 times (*P* < 0.05) in group 1 and by 2.3 times (*P* < 0.05) in group 2, with a significant intergroup difference (*P* < 0.05). A similar trend was observed for the HOMA-IR scores: at T1, a significant 1.66-fold decrease was observed only in group 2 (*P* < 0.05). On T2, a 1.44-fold decrease (*P* < 0.05) was observed in group 1 subjects, and a 2.35-fold decrease (*P* < 0.05) was observed in group 2 subjects, with a significant intergroup difference.

## Discussion

The level of residual cholesterol (or low-density lipoprotein cholesterol, LDL-C) holds significant importance in nonalcoholic fatty liver disease (NAFLD). NAFLD is characterized by the accumulation of fat in liver cells and can progress to more severe conditions, such as nonalcoholic steatohepatitis (NASH), fibrosis, and liver cirrhosis.

The level of LDL-C can be elevated in patients with NAFLD. This is associated with disruptions in lipid metabolism, including increased production and insufficient removal of LDL-C from the liver. Elevated LDL-C levels can contribute to the formation of hepatic fat deposits and inflammatory processes, promoting the development of NASH and disease progression. A study reported a nonlinear association between remnant cholesterol levels and NAFLD [49].

The assertion regarding the positive impact of L-carnitine on the treatment of cholestasis syndrome and its influence on the levels of gamma-glutamyl transferase (GGT) and alkaline phosphatase (ALP) can be justified as follows based on the cholestatic aspect of nonalcoholic fatty liver disease (NAFLD) [50]:

Mechanisms of cholestasis: Cholestasis syndrome is characterized by impaired bile flow from the liver and/or bile ducts. During cholestasis, there is a disruption in the metabolism of bile acids and the accumulation of toxic metabolites, which can lead to elevated levels of GGT and ALP in the blood [51].

Role of L-carnitine: L-carnitine serves as an essential cofactor in the process of beta-oxidation of fatty acids within the mitochondria. In patients with cholestasis, there may be impairment in beta-oxidation of fatty acids and accumulation of lipids in the liver, exacerbating cholestasis and elevating GGT and ALP levels [52].

The action of L-carnitine: L-carnitine can improve the processes of beta-oxidation of fatty acids by facilitating their transport into the mitochondria and providing energy support to liver cells. Additionally, L-carnitine can reduce lipid accumulation in the liver, improve bile flow, and decrease the cholestatic burden on the liver [53].

Clinical studies: Several clinical studies have demonstrated the positive impact of L-carnitine on reducing GGT and ALP levels in patients with cholestasis. For instance, a study published in the journal “Hepatology” in 2011 revealed that supplementation with L-carnitine in patients with nonalcoholic fatty liver disease and concurrent cholestasis resulted in decreased GGT and ALP levels [54, 55].

Based on the aforementioned information, it can be hypothesized that the reduction in GGT and ALP levels observed during the treatment of cholestasis syndrome with L-carnitine may indicate a positive impact of L-carnitine on the cholestatic aspect of NAFLD. However, further research and confirmation in diverse clinical settings and patient populations are required for more specific and compelling conclusions.

In this study, the effect of liver enzymes on the course of NASH showed a more statistically significant effect throughout the study period (90 days) in the experimental group than in the control group. By T1 after the start of treatment, a range of clinical symptoms, such as nausea, bitter taste in the mouth, bowel disorders, and pain in the upper right abdomen, completely disappeared in group 2 subjects who received L-carnitine therapy. After analyzing the dynamics of the main clinical NASH symptoms, we found that therapy involving L-carnitine was more effective than therapy involving essential phospholipids. These findings were supported by the fact that group 2 subjects, who were treated with L-carnitine, had their pain syndrome, bitter taste in the mouth, and bowel disorders completely resolved by T1, with a smaller number of patients complaining of generalized weakness, heaviness in the right hypochondrium, and flatulence, although some patients with NASH did not have these symptoms. As of T2 after the start of treatment, there were more cases of persisting generalized weakness (24.4%), moderate heaviness in the right hypochondrium (20%), flatulence (13.3%), and bitter taste in the mouth (8.9%) among group 1 subjects, who received essential phospholipids, than among group 2 subjects, who received L-carnitine. These data are consistent with the results of other studies that investigated the effects of L-carnitine on the clinical course of NASH [5, 36–38].

During the therapy of nonalcoholic fatty liver disease with L-carnitine, similar to any other medication, there may be potential side effects. However, overall, L-carnitine is considered a safe drug with a low potential for adverse reactions. Despite this, some possible side effects associated with L-carnitine intake include the following:

Gastrointestinal disorders: Diarrhea, nausea, vomiting, abdominal pain, or other symptoms related to the digestive system may occur. However, these symptoms are usually mild and temporary.

Allergic reactions: Some individuals may experience allergic reactions to L-carnitine, such as skin rash, itching, and facial or laryngeal edema. If allergic symptoms occur, immediate discontinuation of L-carnitine intake and consultation with a physician is advised.

Rare side effects: In rare cases, dizziness, headache, increased nervousness, or insomnia may be observed.

The inclusion of phospholipids in the group 1 diet is due to a direct connection of the phospholipids with L-carnitine. L-carnitine therapy causes an increase in the level of phospholipids [37, 56]. Thus, the patients who did not receive L-carnitine but took phospholipids had an initially high level of it. The results of the study confirmed that L-carnitine was more effective than essential phospholipids in terms of liver function normalization, as evidenced by the levels of bilirubin, ALT, AST, ALP, GGT, and thymol turbidity. Administration of L-carnitine in patients with NASH and concomitant obesity exerted the strongest effect on the elimination of cytolytic syndrome by T1 after the start of treatment, with normal values achieved by T2. A more pronounced effect of L-carnitine on cytolysis syndrome (compared to the effect induced by essential phospholipids) is evidenced by a statistically significant intergroup difference (*P* < 0.05) in the activities of ALT and AST in the comparison groups. This positive effect of L-carnitine can be due to its cytoprotective action exhibited through L-carnitine involvement in the synthesis of phospholipids, which are the main components of cell membranes, such as hepatocytes [5, 37]. The results obtained in this study are consistent with those reported by other investigators who have also shown the efficacy of L-carnitine for managing cytolytic syndrome [5, 36, 38, 39].

A decrease in the levels of GGT and ALP confirmed the positive effect of L-carnitine on the management of cholestasis syndrome. In particular, there was a statistically significant decrease in the activities of ALP and GGT at T1 after the start of treatment with L-carnitine, with complete normalization of the ALP and GGT levels by T2. Essential phospholipids turned out to be less effective in managing cholestasis syndrome: a statistically significant decrease in the activities of ALP and GGT was observed only after a 90-day treatment course. In addition, the GGT level measured at this time point was still outside the normal range.

The study also found that the anti-inflammatory effect of L-carnitine therapy was more pronounced than that of essential phospholipids. This statement was evidenced by a decrease in thymol turbidity test values on days 30 and 90 after the start of treatment and the presence of a statistically significant intergroup difference (*P* < 0.05). These data are consistent with other studies dedicated to the investigation of the effects of L-carnitine on liver function tests [5, 39–41, 57, 58].

An analysis of the changes in blood lipids over the course of treatment (Table 3) revealed a positive effect of the prescribed therapies in both comparison groups. However, the therapy involving L-carnitine turned out to be more effective, as evidenced by the presence of a statistically significant intergroup difference for a decrease in the blood levels of TC, TG, LDL, and AI on T2 after the start of treatment, coupled with a statistically significant increase in the content of anti-atherogenic HDL (*P* < 0.05). Thus, this indicates a lipid-lowering effect of L-carnitine. Used in combination with simvastatin, L-carnitine contributed to the optimization of the blood lipid spectrum by T1, and blood lipid levels were almost normalized by T2. These results show that L-carnitine can enhance the lipid-lowering effect of simvastatin. This effect of L-carnitine is due to its participation in the early stages of energy metabolism and lipid catabolism. Its role is the cytoplasmic synthesis of fatty acids since L-carnitine acts as a ‘shuttle’ in the reverse transfer of short-chain acyl groups into hepatocyte cytoplasm across the mitochondrial membrane [5, 37–39, 59].

The changes in the glycemic profile observed during the course of treatment in patients with NASH and concomitant obesity suggest that L-carnitine has a more significant positive effect on carbohydrate metabolism than essential phospholipids (Table 4). This is evidenced by a significant decrease (*P* < 0.05) in fasting and postprandial glucose, decreased insulin levels, and a decrease in the HOMA-IR index by T1 after the start of treatment with L-carnitine. Meanwhile, there was only a tendency toward a decrease in patients receiving essential phospholipids (*P* > 0.05), with a statistically significant difference between the comparison groups (*P* < 0.05). Such a positive effect of L-carnitine therapy on carbohydrate metabolism and IR syndrome has a reason. Thus, L-carnitine, participating in the transportation of free fatty acids across the mitochondrial membrane and their oxidation in the mitochondrial respiratory chain followed by energy release, has a positive effect on the metabolic processes occurring in cells, such as hepatocytes. These effects are a reduction in the liver and muscle concentrations of free fatty acids, an increase in the receptor sensitivity to insulin, and a reduction in hyperglycemia. These data are consistent with those obtained by other authors who studied the effects of L-carnitine on carbohydrate metabolism [37–39, 41, 59, 60]. NASH has a close connection to other factors, such as high uric acid levels. In addition, NASH can also significantly increase the cardiometabolic burden on a patient’s condition starting from childhood [61–63].

### Study strengths and limitations

#### Strengths

The extensive use of random patient sampling for the formation of study groups reduces the likelihood of biases or distortions resulting from the patient assignment.

The utilization of a control group and an experimental group allows for the comparison of results and the assessment of treatment effectiveness.

The study conducted over 90 days enables the evaluation of the long-term effects of therapy using L-carnitine.

The research findings demonstrate a significant improvement in blood lipid profiles, reduction in clinical and biochemical symptoms of non-alcoholic steatohepatitis, and improvement in carbohydrate metabolism.

### Limitations

The study has a limited number of participants (90 patients), which may restrict the generalizability of the results to the broader population.

The absence of long-term follow-up after the completion of the 90-day therapy limits the understanding of long-term outcomes and effects of L-carnitine.

There is a lack of comparison with other treatment methods or placebo control, which may restrict the ability to assess the relative effectiveness of L-carnitine.

## Conclusions

A 90-day L-carnitine therapy in patients with nonalcoholic steatohepatitis and obesity improves clinical symptoms, reduces liver markers (ALT by 3.3 times, AST by 3.0 times), decreases cholestasis markers (γ-glutamyltransferase by 2.2 times, ALP by 2.06 times), and reduces mesenchymal inflammation (thymol test by 3.3 times). It also positively affects blood lipids (total cholesterol decreases by 1.53 times, triglycerides by 2.2 times, low-density lipoproteins by 1.9 times, atherogenic index by 2.8 times, and increases antiatherogenic high-density lipoproteins by 1.47 times). Additionally, it improves carbohydrate metabolism, with fasting glucose decreasing by 14.6%, postprandial glucose by 19.1%, insulin by 2.3 times, and HOMA-IR index by 2.35 times, indicating reduced insulin resistance. More studies, including one by Malaguarnera et al. [31], support the clinical use of L-carnitine, and our paper contributes to the evidence database.

### Prospects for further research

In the future, researchers may focus on the investigation of the features of cytokine homeostasis in patients with nonalcoholic steatohepatitis and concomitant obesity depending on the steatosis degree, the stage of liver fibrosis, and the degree of obesity. Additionally, studies may cover the development of methods for the management of related disorders.

## Data Availability

Data will be available on request to Raisa Aringazina.

## List of Abbreviations

ALT: Alanine aminotransferase
AST: Aspartate aminotransferase
BMI: Body mass index
EASL: European Association for the Study of the Liver
EASL: European Association for the Study of the Liver
EASO: European Association for the Study of Obesity
EMA: European Medicines Agency
FDA: Food and Drug Administration
GH: Growth hormone
HCC: Hepatocellular carcinoma
IR: Insulin resistance
NAFLD: Nonalcoholic fatty liver disease
NASH: Nonalcoholic steatohepatitis
TT: Turbidity test
VLDL: Very-low-density lipoproteins
WHO: World Health Organization
